# Stem Cell-Derived Exosomal MicroRNAs as Novel Potential Approach for Multiple Sclerosis Treatment

**DOI:** 10.1007/s10571-024-01478-1

**Published:** 2024-05-07

**Authors:** Fatemeh Tahmasebi, Elmira Roshani Asl, Zeinab Vahidinia, Shirin Barati

**Affiliations:** 1 Department of Anatomy, Faculty of Medicine, Mashhad University of Medical Sciences, Mashhad, Iran; 2 Department of Biochemistry, Saveh University of Medical Sciences, Saveh, Iran; 3 Anatomical Sciences Research Center, Institute for Basic Sciences, Kashan University of Medical Sciences, Kashan, Iran; 4 Department of Anatomy, Saveh University of Medical Sciences, Saveh, Iran

**Keywords:** Multiple sclerosis, Stem cell, MicroRNAs

## Abstract

Multiple Sclerosis (MS) is an autoimmune disease of the central nervous system (CNS) characterized by inflammation and demyelination of CNS neurons. Up to now, there are many therapeutic strategies for MS but they are only being able to reduce progression of diseases and have not got any effect on repair and remyelination. Stem cell therapy is an appropriate method for regeneration but has limitations and problems. So recently, researches were used of exosomes that facilitate intercellular communication and transfer cell-to-cell biological information. MicroRNAs (miRNAs) are a class of short non-coding RNAs that we can used to their dysregulation in order to diseases diagnosis. The miRNAs of microvesicles obtained stem cells may change the fate of transplanted cells based on received signals of injured regions. The miRNAs existing in MSCs may be displayed the cell type and their biological activities. Current studies show also that the miRNAs create communication between stem cells and tissue-injured cells. In the present review, firstly we discuss the role of miRNAs dysregulation in MS patients and miRNAs expression by stem cells. Finally, in this study was confirmed the relationship of microRNAs involved in MS and miRNAs expressed by stem cells and interaction between them in order to find appropriate treatment methods in future for limit to disability progression.

**Graphical Abstract:**

The effect of miRNAs in transplanted MSC derived exosomes for MS patient treatment. The role of different miRNAs on proliferation, reprogramming, migration and differentiation have been shown.
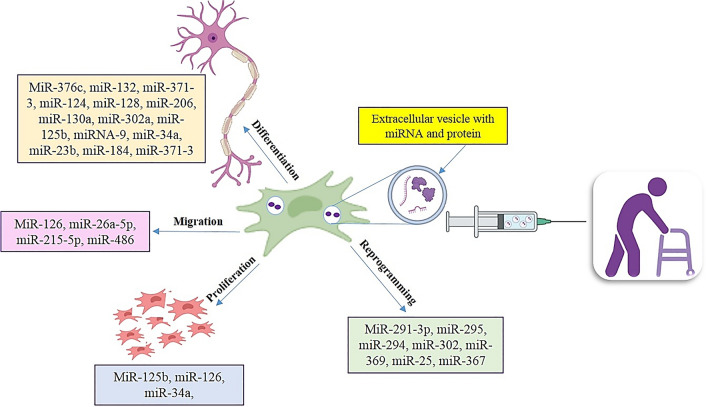

## Introduction

Multiple sclerosis (MS) is a chronic inflammatory and neurodegenerative disease of the central nervous system (CNS), which cause disable young adults around the world. Recently, better understanding of mechanisms involved in pathogenesis of MS helped to the progress of therapeutic strategies for this disease. Thus, it can be really significant to identify biomarkers such as miRNAs that can distinguish Primary-Progressive Multiple Sclerosis (PPMS) from Secondary-progressive multiple sclerosis (SPMS) and progressive MS subtypes from relapsing-remitting MS (RRMS), also predict response to treatments (Sievers et al. 2012). The miRNA expression patterns as disease biomarkers can help to understanding signaling pathways involved in MS. In summary, the aim of the present study was to identify the dysregulation of miRNAs in MS patients and miRNAs expression by stem cells and create a connection between them in order to find appropriate treatment method for limit to disability progression. The findings proposed that circulating miRNAs can be applied as blood biomarkers to diagnose MS. In one hand, dysregulated miRNAs can be considered as diagnostic markers for MS. For that reason, studies on the expression, function, and regulation of miRNAs in MS disease are very important. On the other hand, miRNAs play vital role in viability, proliferation, differentiation, and function of stem cells in damaged conditions. Hence, identification of relation between miRNAs in MS and miRNAs of stem cells can be the therapeutic targets for the treatment of MS in the future. In recent times, reports confirmed that microRNAs have multiple actions in gene regulation and show key roles in most biological processes (Ma et al. 2014). Mesenchymal stem cells (MSCs) are multipotent stromal cells which are extracted from different tissue sources. These cells will be able to self-renew and differentiation into numerous cell lineages such as osteoblasts, adipocytes, chondrocytes, and nerve cells under different conditions (Caplan 2009). Reports showed that MSCs have low immunogenicity and release cytokines and migrate to damaged regions through inflammation and tissue damage, consequently lead to tissue repair and immunomodulation (Rustad and Gurtner 2012). Although MSCs applications for patients are interest for researchers, the molecular mechanisms underlying their self-renewal, differentiation, and regeneration have yet to be completely clarified. Microvesicles (MVs) released from MSCs contain miRNAs can be efficient sources for therapeutic aims (Lakshmipathy and Hart 2008). The researches have revealed that microRNAs, as endogenous post-transcriptional regulators, show a main biological role in modulating proliferation and differentiation of stem cells (Buhagiar et al. 2020). Thus, in the present study, the main objective is the assessment of roles of miRNAs in MS because upregulation and downregulation of various miRNAs may be observed in different phase of MS. Besides MSCs exosomes contain miRNAs which can affected other miRNAs and cells in MS patients. Furthermore, we in this comprehensive review discussed about stem cell-derived exosomal microRNAs as novel potential approach for multiple sclerosis treatment and the deeper understanding signaling pathways of miRNAs action can help to find best therapies for multiple sclerosis patients.

## The Pathophysiology of Multiple Sclerosis

MS is an autoimmune disease of the CNS characterized by inflammation, astrocytosis, microgliosis, and demyelination of CNS neurons leading to cell death and inability that influences millions of individuals around the world (Sospedra and Martin 2016). MS generally is observed in young people and more in women than men, and its chief clinical manifestations include sensory abnormality, limb weakness, visual impairment, and ataxia (Brownlee et al. 2017). In reality, MS is categorized as a multi-factorial disease due to genetic and environmental factors (Mohammed 2020). The environmental factors for instance cigarette smoking, low levels of vitamin D, obesity, and epigenetic alterations have important roles in incidence and progression of this disease (Stampanoni Bassi et al. 2020). Multiple sclerosis has three main clinical subtypes, such as RRMS, PPMS, and SPMS (Ehya et al. 2017). RRMS is the most common type of MS which caused relapses of the disease. At the present time, not only the treatment of MS is vital, but understanding the mechanisms and signaling pathways involves in damage development and MS progression is necessary (Ma et al. 2014). Activated B cells and T cells are seen in the CNS of MS patients. Stimulated T cells activate microglia and macrophages through release chemokines include IL-17 and IFN-γ (Tahmasebi and Barati 2023). M1-activated macrophages help autoimmunity and inflammatory reactions affected by B and T cells, and tissue impairment (Baecher-Allan et al. 2018). These cells help to disease progression and demyelination and axon degeneration (Hemmer et al. 2015). The M1-activated microglia produce pro-inflammatory factors, glutamic acid, and ROS that promote demyelination, inflammation, and neuronal injury (Faissner et al. 2019). Activated peripheral T cells along with infiltrating and brain resident microglia (primary source of innate immune responses) are the major pathogenic reasons in MS (Amoruso et al. 2020).

Because no absolute biomarker is characterized for MS, researchers have frequently evaluated more accurate mechanisms and signaling pathways to discover novel targets for MS disease which can achieve to efficient treatment.

## miRNAs and MS

In the past years, numerous research has been dedicated on finding MS biomarkers that will be able develop to diagnosis the step of disease and improve clinical results. One of targets for study is microRNAs (miRNAs or miRs), little single-stranded non-coding RNAs (19–25 nucleotide length), that modify post-transcriptional expression of mRNAs by either inhibiting their translation or degradation of their structure (Dan Yang et al. 2014). MicroRNAs as a vital subtype of biological molecules have important roles in essential cellular processes such as proliferation, differentiation, and apoptosis in various organs such as brain and immune system (Carthew and Sontheimer 2009). MiRNAs are able to regulate various target genes, influence miRNAs expression and altered biological processes in both brain and immune cells include neurogenesis, neuroinflammation and monocyte/macrophage activation and polarization (Essandoh et al. 2016). MiRNAs can be upregulated or downregulated in different neurological and autoimmune diseases like MS, and therefore, we can distinguish many diseases in initial phase through miRNAs assessment (Abolghasemi et al. 2021).

**Fig. 1:**
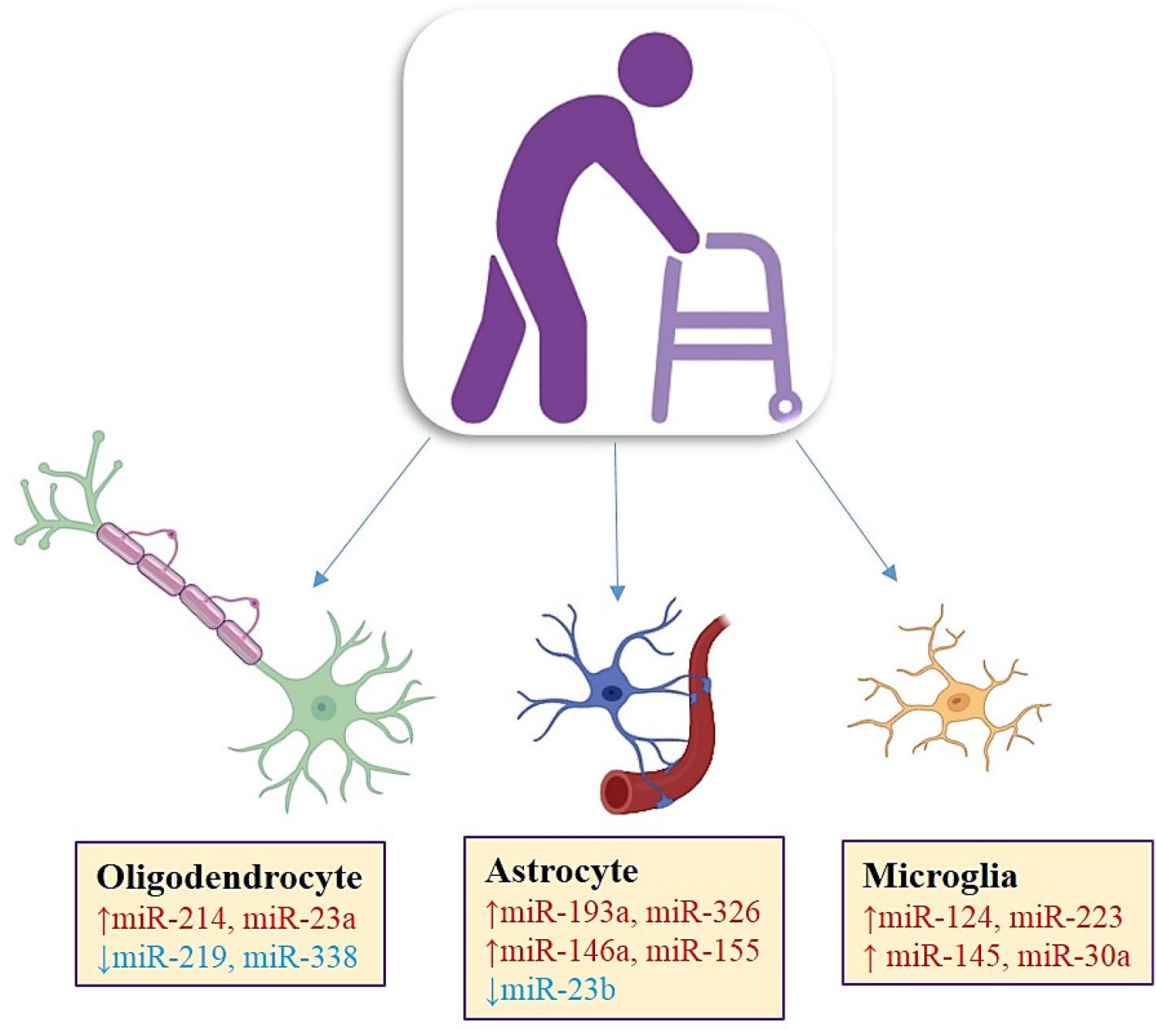
Glial cells related miRNA expression in Multiple Sclerosis. MicroRNA are upregulated (red) and downregulated (blue) in microglia, astrocytes, and oligodendrocytes in the nervous system of MS patients

**Fig. 2:**
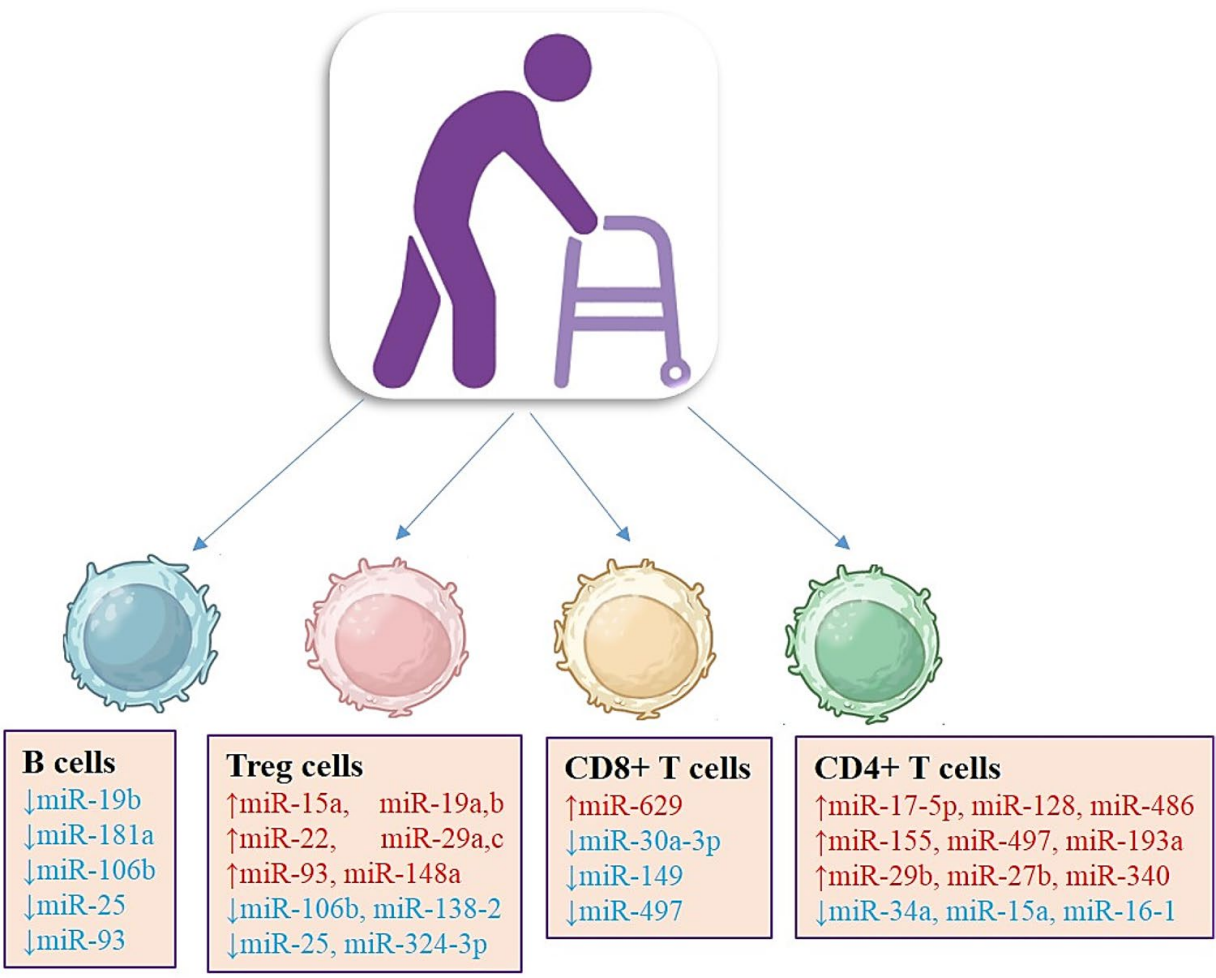
Immune system related miRNA expression in Multiple Sclerosis. MicroRNAs are upregulated (red) and downregulated (blue) in CD4+ T cells, CD8+ T cells, Treg cells, and B cells in the immune cells of MS patients

Several researches have revealed altered expression of miRNA in MS patients (Tarassishin et al. 2011). It is clear that dysregulated miRNAs show an essential role in MS thus, identification of upregulated and downregulated miRNAs in cells of immune system and nervous system can help to distinguish the MS phase and thus found the best approach for MS patients treatment (Søndergaard et al. 2013) (Figs. 1 and 2).

In MS patients, miRNAs were initial reported in blood-derived cells and in active lesions consequently, in the biological fluids, such as cerebrospinal fluid (CSF) and serum (Jagot and Davoust 2016). We summarized the upregulated and downregulated miRNAs in different samples from different phase of MS patients in Table 1.

**Table 1:** Function of miRNAs in MS

MiRNAs	Upregulate	Downregulate	MS phase and target	References
miR-16–2, miR-768-3p, miR-584, miR-664, miR-1275	–		candidate MS biomarkers	(Ghadiri et al. 2018)
miR-9, miR-20b, miR-22, miR-29b-2, miR-30e, miR-96, miR-134, miR-140-3p, miR-150, miR-151-5p, miR-181b, miR-196a, miR-210, miR-337-5p, miR-342-3p, miR-378, miR-431, miR-449b, miR-451, miR-454, miR-500, miR-502-5p, miR-542-5p, miR-574-3p, miR-671-5p, let-7c, let-7d	–		RRMS patients	(Gandhi et al. 2013)
miR-124, miR-24a, miR-10, miR-21	–		As diagnostic targets for early diagnosis of MS	(Ceolotto et al. 2017)
miR-29b, miR-27b, miR-128, miR-340	–		In CD4+ T cells from the peripheral blood of MS patients	(Smith et al. 2012)
miR-1, miR-17-5p, miR-126, miR-193a, miR-200b, miR-376a, miR-485-3p, miR-486, miR-497	–		CD4+ T cells from PBMCs of RRMS patients	(Lindberg et al. 2010)
miR-15a, miR-19a, miR-19b, miR-22, miR-29a, miR-29c, miR-93, miR-106b, miR-107, miR-148a, miR-210, miR-221, miR-223, miR-301a, miR-489, miR590-5p, let-7i	–		In regulatory T cells (Tregs) from peripheral blood samples of MS patients	(De Santis et al. 2010)
miR-629	–		In CD8+ T cells from peripheral blood samples of RRMS patients	(Lindberg et al. 2010)
miR-181c and miR-633	–		In CSF of MS patients	(Ma et al. 2014)
miR-155, miR-338, miR-491, miR-15a, miR-21, miR-22, miR-23a, miR-27a, miR-34a, miR-130a, miR-142-3p, miR-142-5p, miRNA-146a, miR-146b, miR-193a, miR-199a, miR-200c, miRNA-214, miR-223, miR-320, miR-326 and miR-650	–		In the active brain white matter lesions from patients with MS	(Junker et al. 2009)
miR-146a-5p	–		in RRMS and PPMS monocytes	(Pauley et al. 2009)
miR-422a, miR-572, miR-614, miR-648, and miR-1826	–		in plasma from patients with MS	(Siegel et al. 2012)
miR-660, miR-939, miR-376a-3p, miR-21, miR-146a and –b	–		in plasma from patients with RRMS	(Søndergaard et al. 2013)
let-7c, miR-92a-1, miR-135a, miR-145, miR-454, miR-500, and miR-574-3p	–		in plasma from patients with SPMS	(Gandhi et al. 2013)
miR-128-3p, miR-376c-3p, miR-24-3p	–		PPMS	(Guerau-de-Arellano et al. 2011)
miR-191-5p	–		in SPMS and PPMS	(Baulina et al. 2018)
miR-21-5p, miR-124-3p	–		MS	(Abolghasemi et al. 2023)
miR-10, miR-124, miR-193b-3p		–	in RRMS	(Baulina et al. 2018)
miR-145		–	in plasma from patients with RRMS	(Søndergaard et al. 2013)
miR-15b, miR-23a,		–	in serum of RRMS and PPMS patients	(Fenoglio et al. 2013)
miR-23b, miR-139, miR-181c, miR-184, miR-328, miR-340, miR-487b, miR-656		–	white matter tissues	(Junker et al. 2009)
miR-922, miR-20a		–	in CSF of MS patients	(Ma et al. 2014)
miR-30a-3p, miR-149, miR-497		–	in CD8+ T cells from peripheral blood samples of RRMS patients	(Lindberg et al. 2010)
miR-138–2, miR-324-3p, miR-338-5p, miR-512-3p, miR-564, miR-886-3p		–	in regulatory T cells (Tregs) from peripheral blood samples of MS patients	(De Santis et al. 2010)
miR-34a		–	CD4+ T cells from PBMCs of RRMS patients	(Lindberg et al. 2010)
miR-15a, miR-16–1		–	in CD4+ T cells from peripheral blood samples RRMS patients	(Jernås et al. 2013)
miR-15b, miR-23a, miR-30c, miR-99b, miR-125a-5p, miR-150, miR-197, miR-320d, miR-339-5p, miR-361-5p, miR-423-3p, miR-423-5p, miR-494, miR-625, miR-663, miR-1260b, miR-1280, miR-1979, miR-3153, miR-3178, let-7a		–	T cells from peripheral blood samples of MS patients	(Ma et al. 2014)
miR-1979, miR-7–1, miR-144, miR-15a, miR-16, miR-17, miR-20a, miR-20b, miR-27a, miR-27b, miR-93, miR-98, miR-106a, miR-126, miR-140-5p, miR-211, miR-374a, miR-510, miR-579, miR-623, miR-624, miR-20a-5p, miR-7–1-3p, let-7d, let-7f, let-7 g, let-7i		–	all MS subtypes including primary progressive, secondary progressive and relapsing–remitting disease	(H. Wang et al. 2016a, b)
miR-92, miR-135b, miR-153, miR-189, miR-422a		–	in B cells from peripheral blood samples of RRMS patients	(Lindberg et al. 2010)
miR-7–1, miR-15a, miR-16, miR-19b, miR-25, miR-28-5p, miR-93, miR-103–2, miR-106b, miR-107, miR-130b, miR-140-5p, miR-151-5p, miR-152, miR-181a, miR-191, miR-200a, miR-200b, miR-203, miR-204, miR-218, miR-221, miR-297a, miR-299a-5p, miR-320b, miR-329, miR-337-3p, miR-340, miR-363, miR-369-5p, miR-411, miR-486-5p, miR-515, miR-520a-3p, miR-520 g, miR-548c-5p, miR-551a, miR-582-5p, miR-585, miR-599, miR-616, miR-624, miR-644, miR-649, miR-655,let-7i		–	B lymphocytes from peripheral blood samples of RRMS patients	(Sievers et al. 2012)

### Function of miRNAs in MS Pathology

In the recent decade, researchers could use MiRNAs as MS diagnostic markers. Most MS-associated miRNA reports are attentive on miRNA expressions in blood mononuclear cells, B cells, and various populations of T (CD4+ and CD8+), but less data are obtainable on circulating miRNAs (Kacperska et al. 2015). In one paper, over 900 miRNAs were described from plasma of 4 MS patients (Siegel et al. 2012). Other study showed that 8 of 368 miRNAs were expressed in plasma of SPMS compared to RRMS patients (Gandhi et al. 2013). Relapsing and progressive subtypes of MS have different underlying pathogenic mechanisms and biomarkers (miRNAs) that express the processes and phase of disease. The studies showed that in MS patients, miR-29b increased in memory CD4+ T cells from the peripheral blood, and miR-29b expression was repressed considerably upon T-cell activation in MS (Ponomarev et al. 2013). In one research, miR-155 expression as pro-inflammatory factor significantly upregulated in peripheral circulating CD14+ monocytes of MS samples (Moore et al. 2013). A set of miRNAs was expressed that regulate monocyte-macrophage activation in clinical remission of MS patients and help to define the activation state of these cells in RRMS and PPMS samples (Kacperska et al. 2015). MiR-124 as pro-regenerative factor maintains brain homeostasis via regulation of neurogenesis, synapse plasticity, and glia-neuronal interactions. The researches representative of miR-124 are highly expressed in resting microglia (Svahn et al. 2016) and miR-124 downregulation has been related with neuroinflammatory disorders including dementia, PD, and MS (H. Wang et al. 2016a, b). Monocytes/macrophages are major immune cells responsible for cellular pathology and tissue injury in MS (Blonda et al. 2017). Significantly, the miRNAs can influence macrophage and microglia activation besides regulation of neuron and oligodendrocyte activity and finally synaptic transmission and remyelination (Prada et al. 2018). The reports have been indicated that downregulated miRNAs can target the genes encoding the B cell receptor, phosphatidyl-inositol-3-kinase (PI3K), and phosphatase and tensin homology signaling pathways (Sievers et al. 2012). Also, miR-128 is upregulated in CD4+ T cells of RRMS, SPMS, and PPMS patients (Guerau-de-Arellano et al. 2011). MiR-128 suppresses Th2 cell differentiation and promotion of pro-inflammatory Th1 responses and do not have a role in the neuronal proliferation, differentiation, and apoptosis (Adlakha and Saini 2014). Recently, a report indicated that miR-24-3p suppress interferon-γ (IFN-γ) expression via direct binding to its mRNA target regions (Fayyad-Kazan et al. 2014). Furthermore, IFN-γ is secreted from Th1 cells, and upregulation of miR-24-3p leads to maintain immune system homeostasis and development of regulatory responses in PPMS. Interestingly, both miR-128-3p and miR-24-3p control mechanisms involve in MS pathology such as ErbB and p53 signaling pathway, T-cell receptor pathway, and ubiquitin-mediated proteolysis (Giacalone et al. 2015). Also, miR-191-5p upregulated both PPMS and SPMS and regulates cellular differentiation and innate immune responses (Hemmer et al. 2015). Downregulation of MiR-191 in B lymphocytes of untreated RRMS patients shows its role in the B-cell-mediated immune responses (Sievers et al. 2012). However, miR-376c is extremely expressed in neurons in order to neuronal differentiation. Interestingly, miR-376c-3p was upregulated in PPMS (Jovičić et al. 2013). The one study showed reduced levels of transforming growth factor-β (TGF-β) in MS patients. Also, TGF-β signaling suppression has a vital role in the MS pathogenesis by regulating Th17 and Treg responses (Liu et al. 2014). The abnormal expressions of circulating miRNAs were related mostly with PPMS proposing their contribution in immunopathogenesis of PPMS (Meoli et al. 2011).

Additionally, there is disagreement between circulating miRNAs reported, due to differences in patient cohorts, miRNA study methodologies, and statistical approaches, lacking protocols for miRNA research. Parallel to these results, there is a report that miR-21 and miR-124 are deregulated in autoimmune diseases, such as MS, contribute in their pathogenesis through neuroinflammation pathway (Gaudet et al. 2018). The upregulation of miR-15a, miR-223, and miR-19a was confirmed in both Treg cells, blood cells, plasma samples, and brain white matter tissues from MS patients, suggesting that these have a role in MS pathogenesis. Also, downregulation of miR-328 (in brain white matter lesions), miR-15a, and miR-15 (in blood, peripheral T cells and B cells or plasma samples) was observed in MS samples, emphasizing that these reduction of miRNAs have consequences in MS pathogenesis. Especially, miR-15a was increased in brain white matter lesions and Treg cells, but decreased in blood, B cells, and peripheral T cells, showing that regulation of miRNA expression in MS was under discussion (Raposo and Stoorvogel 2013) (Fig. 1).

### Regulation of miRNAs in MS

The findings recommended that cytokines and drugs can regulate miRNA expression in MS. Interferon-beta (IFN-β) is largely applied in the MS treatment. The results revealed that miR-342-5p, miR-16-5p, miR-346, miR-760, miR-518b, let-7a-5p, and let-7b-5p were upregulated, while miR-29a-3p, miR-27a-5p, miR-29b-1-5p, miR-95, miR-29c-3p, miR-149-5p, miR-193a-3p, miR-181c-3p, miR-193-5p, miR-532-5p, miR-423-5p, miR-874, and miR-708-5p were downregulated in MS samples after IFN-β administration, that was associated with IFN feedback circles and apoptotic processes (Hecker et al. 2013). In addition, IL-17 increased in MS patients (Pöllinger 2012). Studies showed that IL-17 inhibited miR-23b expression in the spinal cords of MS mice (EAE model), and also miR-23b reduced IL-17 (Zhu et al. 2012). Glatiramer acetate, an immunomodulation drug for MS, significantly decreases miR-142-3p and miR-146a expression in MS patients (Waschbisch et al. 2011). Natalizumab as a monoclonal antibody against α4-integrin is administrated to MS patients (Yaldizli and Putzki 2009). In one research, miR-19b, miR-142-5p, miR-106b, miR-150, miR-383, miR-191, miR-598, and miR-551a were increased in B lymphocytes of peripheral blood samples from RRMS patients after natalizumab treatment for six months compared with untreated RRMS patients (Sievers et al. 2012). The findings propose that non-immune modulated drugs can control miRNA expression in MS and thus miRNA adjustment can be a therapeutic approach for MS patients.

### MiRNAs as MS Therapeutic Targets

The researches indicated which upregulation of miR-155 and miR-326 is observed in MS patients (Zhang et al. 2014) inhibition of miR-326 and miR-155 expression could suppress Th1 and Th17 cells, subsequent the reduction of disease progression in EAE model mice of MS. Additionally, anti-miR-155 and miR-326 administration considerably repressed disease progression and can be a good therapeutic object for MS (Murugaiyan et al. 2011). This is showing which miR-23b can be an appropriate target for treatment of MS through IL-17 reduction, suppressed tumor necrosis factor alpha (TNF-α)- or IL-1beta-induced nuclear factor kappa-B (NF-κB) activation, and inhibit TGF-β-activated kinase 1/MAP3K7 binding protein 2 (TAB2), TAB3, and NF-κB kinase subunit alpha (IKK-α) in EAE mice (Zhu et al. 2012). One study revealed which reduction of miR-125a-5p contributes in the BBB damage related with MS, and accordingly miR 125a-5p can be applied in the BBB function recovery for efficient therapy of MS (Reijerkerk et al. 2013). The results exhibited the upregulation of miR-124 expression in the demyelinated brain *hippocampi* from MS patients, that indicate miR-124 have a significant role in the MS pathogenesis and miR-124 regulation can help to MS treatment (Ma et al. 2014). It has been informed that miR-27b, miR-340, and miR-128 were increased in CD4+ T cells from MS patients and inhibit IL-4 expression, shift Th2 to Th1, suppress Th2 development, and leading to MS pathogenesis. Thus, miR-27b, miR-340, and miR-128 inhibitors can lead to renew Th2 responses in T cells from MS patients and miR-27b, miR-340, and miR-128 suppression is suggested in MS treatment (Guerau-de-Arellano et al. 2011). The reports have shown that miR-21, let-7e, and miR-155 stimulate Th1 and Th17 development, miRNA-21 rises the of IFN-γ and IL-17 expression, miR-146a upregulates IL-17 expression, miRN-326 helps Th17 development, and miR-142-3p prevents IL-10 expression, which help to MS pathogenesis (Arora et al. 2013).

## Cell Therapy Strategies for Multiple Sclerosis Treatment

In the first part of this study, we review recent insights into the upregulation and downregulation of miRNAs in MS. In the second part, we will discuss about important role of miRNAs in stem cell differentiation and function after MSCs transplantation (Xu et al. 2009). Therefore, understanding of the role of miRNAs in MS pathology and stem cells regulation and establish communication between them can open the window toward achieve to novel therapeutic approach for neurodegenerative disease (Jakob and Landmesser 2012).

Fundamentally, MS therapy approaches such as drugs, normally used at various stages of MS, can regulate and repress the inflammation and neurodegeneration of CNS but there is no evidence to prove their effects on the progress of repair activity or axon remyelination. Stem cells with immunomodulatory properties and multilineage differentiation ability are listed as a beneficial therapeutic approach in neurodegenerative diseases such as MS (Barati et al. 2020). Stem cells are undifferentiated cells which can differentiate to various lineage cells. Mostly, they are categorized into embryonic stem cells (ESCs), pluripotent stem cells (iPSCs), and adult stem cells (ASCs). Based on the originated tissue, they are known mesenchymal stem cells, neural stem cells (NSCs), hematopoietic stem cells (HSCs), and endothelial stem cells (Takahashi and Yamanaka 2006).

Stem cells were used in clinical therapy for different diseases because they have self-renewal, pluripotency, and differentiation ability.

## The miRNA Expression in MSCs and Microvesicles

Stem cell-derived extracellular vesicles are more appropriate for cell therapy than cells because are smaller and less complex, have not got engraftment limitations, short viability, stem cells differentiation into unwanted cell lineages and risk of tumorigenicity, and ethical restrictions (Wei et al. 2021). The identification of the cellular source of exosomes is very important (Viñas et al. 2018). Recent evidences revealed that exogenous MSC transplantation has beneficial effects at the site of injury in different diseases that suggesting MSCs have a paracrine support to the repair (Humphreys et al. 2008). Exosomes are 40–100 nm vesicles consisting of membrane and cytosolic proteins, lipids, and RNA which reflect their cell of origin and can cross the BBB and facilitate brain regeneration after damages (Haney et al. 2015). These vesicles reflect the biological information of source cells and contribute in cellular crosstalk between adjacent cells in the damage area under physiological and pathological situations (Peng et al. 2022). The exosomes mediate cell–cell communication through autocrine and paracrine function and control cellular processes, including development, inflammation and immune responses, metabolic diseases, neurodegeneration, and cancers (Dai et al. 2020). The administration of the best exosome-released cells can increase the benefit effects of exosomal application (Xu et al. 2022).

Nowadays, microvesicles are mostly used instead of stem cells because function, homing, differentiation, and viability of BMSCs are impairment when extracted from aged patients with chronic disease (Chavakis and Dimmeler 2011). Recently have been displayed that MSCs derived MVs which are spherical fragments of membrane secreted from the cells as exosomes (Camussi et al. 2010). The studies demonstrate that MSC exosomes contain a characteristic proteins, mRNAs, and miRNAs (Smalheiser 2007). Hence, MSCs have capability for miRNA-mediated biological effects on other cells via release of pre-miRNA in exosomes (Chen et al. 2010). It has been documented that on the one way, MSCs stimulate tissue regeneration through growth factors, cytokines, and extracellular matrix molecules secretion and also transfer of genetic information between MSCs and cells of tissue damaged. On the other way, MVs released by MSCs contain miRNA that prompts cell reprogram, viability, dedifferentiation, and migration and finally tissue regeneration (Camussi et al. 2010). Latest evidence indicates that miRNAs have critical role in control of physiological functions, such as immune response, hematopoiesis, stem cell differentiation, and neurogenesis (Tay et al. 2008). The studies showed that over 40% to 90% of the human protein encoding genes are regulated by miRNA-mediated genes (Hu et al. 2010). Recently, reports revealed that miRNAs are main regulators of stem cells via directly targeting the 3′UTR of pluripotency factors and coding sites of transcription factors to moderate stem cell differentiation and therefore can change the fate of stem cells in various diseases (Tata et al. 2011). Essentially, microRNAs are produced by brain cells and flow in body fluids bound to RNA-processing molecules reveal alterations of the brain conditions (Kimura et al. 2018) (Fig. 1). MiRNAs extracted from blood or brain samples of MS patients have many advantages as a diagnostic markers and providing data of the activated immune cells in CNS of patients and consequently help to beneficial therapy. Furthermore, specific miRNAs have been controlled cellular development and differentiation nervous system (Gangaraju and Lin 2009). MSCs express surface receptors including CD73, CD105, CD90, and CD29 and the absence of hematopoietic markers such as CD34, CD79a, CD45, CD19, CD11b, and CD14 marker (Dominici et al. 2006). Additionally, MSCs have immunoregulatory properties that can rise T regulatory cells and to suppress the proliferative response of antigen-specific memory T cells in vitro (Crop et al. 2009).

As mentioned above, MicroRNA as a type of small non-coding single-stranded RNA which acts on the 3’UTR of target genes, inhibit the translation of target gene RNA, make it degrade to show a regulatory role in stem cell viability, differentiation, renewal, cell cycle, and apoptosis and hence have a significant role in physiological and pathological processes (Han and Fan 2021). Recently, reports show that miRNAs have a main role in stem cell preservation, activities, and differentiation. As well, several genes were discovered to selectively related with unlike stem cell populations according to their origin (Gangaraju and Lin 2009). The evaluation of miRNA between pluripotent, multipotent, and somatic stem cells shown specific miRNAs involved in the regulation of cell differentiation. The stem cell lines were classified into two groups included the ESCs (pluripotent) and the ASCs (multipotent) after 250 mature human miRNAs analysis (Aranda et al. 2009). There are 64 different miRNAs between ESCs and ASCs which 34 miRNAs are upregulated and 30 miRNAs downregulated. The ASCs are divided into two populations including the adult progenitors and the MSCs with few different miRNAs which miR-143, miR-199b, and miR-129 are downregulated and miR-424 and miR-204 are upregulated in adult progenitors with respect to MSC (Aranda et al. 2009). Just few miRNAs play a functional role in developmental processes for instance miR-181a stimulates the differentiation of hematopoietic stem cells into B cells (Chen et al. 2004). miR-134 controls the development of dendritic spine hence is considered specific for brain. The suppression of miR-371-3 could stimulate neural differentiation (Schratt et al. 2006). Similarly, miR-124 is identified in the M2 microglia-derived exosomes, that can be used to suppress neuronal autophagy and inflammation, develop neurogenesis, and improve repair after brain diseases (Ge et al. 2020). The miRs regulate cell renewal, reprogramming, differentiation, and block apoptosis control adult stem or progenitor cells activity (Leri et al. 2011). The specific miRs facilitate stem cells differentiation to specific cell lineages and change cell fate through reprogramming of adult progenitor cells (Heinrich and Dimmeler 2012).

### The Effect of miRNAs on Cell Viability and Function

Cell viability was controlled by numerous miRNAs in adult progenitor cells. MiRs control gene expression in different cellular processes. For instance, miR-126 increased viability and migration of MSCs and therefore upregulate the efficiency of transplanted cells (Huang et al. 2013). One paper showed that miR-126 upregulation can increase the paracrine factors secretion from MSC, that help to the regeneration after cell injection (Huang et al. 2013). The exosomes contain miRNAs which mediate cell-to-cell communication between numerous cell types (Hergenreider et al. 2012). MiR-34a represents cells survival and the functional capacity that play a significant role in disease treatment after cell therapy (Xu et al. 2012). As well, miR-34a regulated apoptosis signaling through antiapoptotic protein B cell lymphoma 2 (Bcl-2), protects MSCs from apoptosis, and it suppresses cellular proliferation by inhibition of cell cycle regulators (Ieda et al. 2010). Stimulation of miR-146a prevented from apoptosis via a nuclear factor-kB-dependent. The miR-146a displays protective role by suppression of Fas (CD 95) as a tumor necrosis factor receptor family member, which control cell death reprogramming (Suzuki et al. 2010).

### The Effect of miRNAs on Reprogramming

**Fig. 3:**
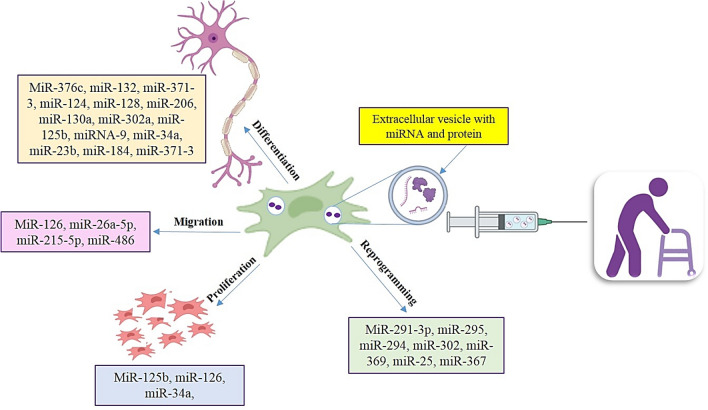
The effect of miRNAs in transplanted MSC-derived exosomes for MS patient treatment. These extracellular vesicles contain microRNAs and proteins. The role of different miRNAs on proliferation, reprogramming, migration, and differentiation has been shown

The studies propose that the controlled expression of cell cycle regulators can help to the reprogramming improvement through the miRNAs (Judson et al. 2009) (Fig. 3). MiR-291-3p, miR-295, miR-294, miR-302, miR-369, and miR-367 augment the efficiency of reprogramming, while the miR-292-3p and miR-293 were not effective (Judson et al. 2009). The miR-294 overexpression can increase the reprogramming efficiency to 75% (Judson et al. 2009). The miRs may regulate reprogramming by targeting of TGFβ signaling pathways. For example, the miR-25 targets the TGFβ receptor-2 and miR-302/miR-372 inhibited TGFβ-induced epithelial–mesenchymal transition, proposing that suppression of TGFβ can have a vital role in reprogramming by miRs (Subramanyam et al. 2011). This result is showing that TGFβ receptor silencing or repression leads to increase reprogramming, while TGFβ activation decreases reprogramming (Li et al. 2011). Finally, miRs can be a safe and efficient route for viability, proliferation, differentiation, and migration of stem cells either inhibit or prompt reprogramming (Choi et al. 2011).

### The Role of miRNAs in MSC Differentiation

The miRNA expression is different in embryonic and adult stem cell which showing functional importance of miRNA during development and differentiation (O'Rourke et al. 2007) (Fig. 3). One of the studies have been reported, specific miRNAs are involved in the differentiation of MSCs into other lineages cell such as neurons and MSC-derived neurons release neurotransmitter and synaptic plasticity (Greco et al. 2007) (Fig. 3). For instance, miR-124 and miR-9 induce neurogenesis (Yoo et al. 2009). The let-7 family of miRNAs has important role in differentiation and the self-renewal of MSCs (Viswanathan et al. 2008). The pluripotent markers include miR-200c, miR-302a, b, c, and d are absent in MSCs (Peter 2009). MiR-125b controls both cell proliferation and differentiation. Also, MiR-125b inhibited astrocyte-specific mRNAs during neural precursor expansion and differentiation in stem cell-derived neural progenitor (Mizuno et al. 2008). A histone 3 lysine 9 demethylase maintains miR-302 expression and inhibit the neural differentiation of ESCs (Wang et al. 2014). The miR-124 and miR-128 expression induce neuronal cell differentiation (Smirnova et al. 2005). The miRNAs have potential in the neuronal-like cells generation. A research recognized upregulation of 16 miRNAs after differentiation. The miR-206, miR-130a, and miR-302a significantly increased in MSC-derived neuronal-like cells compared with undifferentiated BMSCs (Greco and Rameshwar 2007). The miR-125a, miR-125b, miRNA-9, and miR-23b were identified in undifferentiated BMSCs that are involved in neural differentiation (Greco and Rameshwar 2007).

The numerous studies have confirmed to the role of miRNAs in neurogenesis. The miR-124 stimulates the neural differentiation of the subventricular zone, which is the largest neurogenic niche in adult brain (Cheng et al. 2009). The upregulation of miR-34a expression leads to reduction of dendritic length, neuron branch numbers, and the numbers of functional synapses (Agostini et al. 2011). miR-9 promotes neural stem cell differentiation (Zhao et al. 2009). Furthermore, increased miR-184 regulates adult NSC differentiation which can control the brain development (Liu et al. 2010). MiR-371-3 is vastly expressed in ESCs and iPSCs and miR-371-3 suppression helps neural differentiation (Kim et al. 2011). The reduction of miR-132 induces the neuronal differentiation (Dehua Yang et al. 2012). miR-125 can promote stem cells differentiation to the neural cells (Boissart et al. 2012) and miR-302 can form a regulation loop during neural differentiation (Rosa and Brivanlou 2011). MiRNAs can help the progress of regenerative medicine in the future through reprogramming process, pluripotency maintenance, and differentiation of stem cells. Additionally, the major function of miRNAs in the determination of stem cell fate can be the route for treatment neurodegenerative diseases (Shah et al. 2010).

## MiRNAs Expression Among MSCs Derived from Different Tissues

As mentioned above, MSCs with pluripotent-like properties are a kind of adult stem cells that have been derived from different tissues (Aquino et al. 2010). Several studies point to that MSCs have low immunogenicity and release cytokines and migrate to damaged area in inflammation and tissue injury, consequently lead to control inflammation and regeneration (Rustad and Gurtner 2012).

Tsai et al. (2007) evaluated the transcriptome expression of MSCs derived from bone marrow, cord blood, amniotic membrane, and fluid (Tsai et al. 2007). The results show that MSCs derived from different organs have a common pattern of miRNAs, specific of MSC lineage. The transcriptomes of MSCs from different organs are to recognize the principal feature of all MSCs and the gene expression profile of each MSC population (Tsai et al. 2007). Assessment of the miRNA expression profiles on ADSCs and BMSCs shows that the only difference is downregulation of the miR-424 expression in BMSCs (Aranda et al. 2009). Moreover, other study showed eight miRNAs expressed by MSCs (miR-196a and b, miR-615, miR-501, miR-449, miR-17-3p, miR-497, and miR-486) (Collino et al. 2010). MicroRNAs can modulate MSC phenotype, change their fate, and regulate the crosstalk of MSC with tissue-resident cells in order to tissue repair.

## Therapeutic Effects of Stem Cell‑Derived Exosomal miRNAs

### The Effects of Adipose-Derived Mesenchymal Stem Cells Exosomes on miRNAs of MS Patients

There are researches that indicate ADSCs can improve many diseases and also have protective effects against the damage through releasing exosomal miRNA. One report showed that ADSC-derived exosomes secrete a high level of miR-26a-5p which could reduce cell apoptosis by inhibiting the mTOR signaling activation, downregulate VEGFA, increase viability, and silencing the NF-κB pathway (Duan et al. 2020). In addition, ADSCs-exosomes can transport miR-26a-5p, miR-215-5p, and miR-486 to cells, therefore reduce cell apoptosis and control migration, thereby regulate cellular function (Hao et al. 2021). Exosomes released from ADSCs can inhibit the IL-6 expression. Also, ADMSC-exosomes release miR-125a which have protective effects and the reduction of miR-125a repressed the protective effects of ADMSC-exosomes that have an important role in neurodegenerative diseases (Liang et al. 2016). ADSC-derived exosomes secrete miR-486 that decrease the expression of Smad1, thus inhibit the mTOR pathway activation and cell apoptosis (Jin et al. 2019).

### The Effects of Bone Marrow Mesenchymal Stem Cells (BMSCs) Exosomes on miRNAs of MS Patients

There are two main stem cell populations in bone marrow include the hematopoietic stem cells, which differentiated into blood cells and the mesenchymal stem cells, which derived from bone, cartilage, fat, and muscle (Jiang et al. 2002). Furthermore, stem cells can differentiate into cells of their tissue of origin and lead to tissue repair (Phinney and Prockop 2007). BMSCs have been used in cell therapy for different neurodegenerative diseases such as MS (Sun et al. 2018). Especially, exosomes secreted from BMSCs transfer of miR-222 that reduce TGF-β expression via the downregulating STAT5 and improvement damages (Gallo et al. 2016). One study has been shown that exosome derived of BMSC contains miR-let-7a significantly improved functional parameters (Mao et al. 2020). Interestingly, the report indicated that miR-125b in BMSC-Exosomes can remarkably inhibit apoptosis via suppressing TGF-β1 signaling pathway. Also, miR-125b and miR-let7c secreted of BMSCs protect cells and thus improve injury (B. Wang et al. 2016a, b).

Similarly, miRNAs were identified in vesicles from MSCs play a critical role in tissue regeneration (Zhao et al. 2019). The results indicated 21 miRNAs were upregulated in exosomes. The upregulation of miR-29a and miR-381 reduces M1 macrophages (pro-inflammatory phenotype) and suppresses TGFb1 and therefore induces tissues regeneration. Findings revealed that miR-29a and 381-5p have anti-inflammatory effect and suppressed iNOS, CXCL10, and MCP1 expression after LPS treatment (Hsia et al. 2016). The miR-381 regulates inflammation via NFkB signaling and control IL-6, TNFα and COX-2 expression. Exosome transplantation has not got limitation and increases clinical effectiveness of the cell therapy (Xu et al. 2015).

## Limitations of MSC Therapy for the Treatment of MS Patients and Future Perspectives

Cell therapy is one of the treatment approaches for MS. Although this method is suitable for nerve and immune repair modulation in MS, no definitive therapeutic outcome has been reported so far. Cell therapy, like other methods, has several limitations, but it is superior to other treatment methods. Because MSCs secrete cytokines and are capable of immune modulation, repair, and remyelination. There are still obstacles that require studying the limitations and mechanisms of this treatment. The course or stage of the disease is one of the major problems. Patients may show different responses to the treatment process. It is especially important in cell therapy, because they require proper treatment.

Injection method and sufficient amount of cells for optimal outcomes are necessary. The cell source of the graft is of great importance. Among the different populations of stem cells, the best cell type must be selected. For example, cells such as MSCs can be derived from different sources for injection, each with specific advantages and disadvantages. Isolation and culture should also be accurate and follow standard protocols to minimize side effects in patients. In addition, it requires evaluation of toxicity, microbiological, mutagenesis test, and type of phenotype and karyotype (Boissart et al. 2012). Stem cell-derived exosomes (contain miRNAs) transplantation is better than MSCs transplantation because they are smaller and less complex, have not got engraftment limitations, short viability, stem cells differentiation into unwanted cell lineages, and risk of tumorigenicity and ethical restrictions. When we measure miRNAs in peripheral blood of MS patients, we can distinguish MS phase and used appropriate source of stem cells (according to present miRNAs) for transplantation.

Small sample size and the lack of a suitable control group are often a major drawback in human samples. In some studies, when the desired results are not achieved or the recovery rate is slow, it may be necessary to repeat the cell injection. However, sometimes patients cannot be convinced to accept the conditions for such a procedure (Abolghasemi et al. 2023). If the patients know that stem cell-derived exosomes injection is safer than MSCs injection, they interest this approach for treatment.

The method of injection and administration of cells is very important. Difficulty in linking the heterogeneity of the disease and the multifocal nature of MS lesions can limit the effectiveness of an injection method. For example, in human samples, intravenous procedures are more common because intrathecal procedures may cause meningeal injury. In addition, local injection methods cannot be used in the demyelinated area such as corpus callosum or intraventricularly. However, intravenous injection results in a large amount of cells being trapped in the lungs and other tissues, which reduces the success and effectiveness of cell therapy. Concerns about microembolization have precluded the use of MSCs transplantation (Boissart et al. 2012). In addition to the appropriateness of the injection method, the number of cells per injection and the frequency of injection are also important. In many human studies, the number of cells is either too low to assess the effect of cell therapy on patient recovery, or with repeated injections too high to be fatal or cause other disorders and pathological conditions. The details of survival, migration, and differentiation mechanisms of stem cells injected into damaged areas in clinical trials are unclear (Collino et al. 2010). Stem cell-derived exosomes can be injected high number because have not got MSCs number injection limitation.

Stem cell-derived exosomal miRNAs are able to reduce the CNS damage with immunomodulatory effects, by suppressing dendritic cell and lymphocyte function and reducing gliosis. These mechanisms are very complex and require extensive study, and therefore, their tracking is of particular importance in future.

Several studies have established that miRNAs have vital roles in physiological and pathological processes in different diseases including myocardial injury and neurodegenerative, muscle, and blood damages (Barwari et al. 2016). Studies show that the great potential of stem cell-derived exosomal miRNAs for the treatment of MS is due to their immunosuppressive and immunomodulatory properties, as well as myelin improvement and neuroprotection. But many questions about the mechanism of action of them in humans are unanswerable. One of the challenges is some miRNAs were upregulated in a MS phase but they downregulate in other MS phase. In future, we can assess more accurate mechanisms of miRNAs action in diseases that achieve to methods for MS treatment by using specific miRNAs.

## Conclusion

Finally, this study discussed dysregulation of miRNAs can play an important role in pathogenesis of different phase of MS and can applied as diagnostic target and one of new screening approaches for RRMS and monitoring of the disease progress. Attention to our finding, we can assessment miRNAs level in MS patients and after diagnosis of MS phase, patients will receive the best therapy. On the other hand, circulating exosomal miRs assessment after cell therapy reveals that numerous downregulation of biomarkers in MS patients could reflect the therapeutic efficacy of the cell therapy. Moreover, this study assessed miRNAs expression in stem cells and their effects on miRNAs of MS patients. We studied the relationship of miRNAs involved in MS and miRNAs expressed by stem cells and interaction between them in order to find appropriate treatment method for limit to disability progression.

Our finding showed that understanding of underlying mechanism in these connections can lead to achieve beneficial approaches for MS treatment.

## Data Availability

Enquiries about data availability should be directed to the authors.

## References

[CR1] Abolghasemi M, Poursaei E, Bornehdeli S, Shanehbandi D, Asadi M, Sadeghzadeh M, Sadeh RN (2021) Exploration of potential circulating micro-RNA as biomarker for Alzheimer’s disease. Meta Gene 30:100968

[CR2] Abolghasemi M, Ali Ashrafi S, Asadi M, Shanehbandi D, Sadigh Etehad S, Poursaei E, Shaafi S (2023) MicroRNAs expression in peripheral blood mononuclear cells of patients with multiple sclerosis propose. Mol Biol Rep 50(1):167–172 36319782 10.1007/s11033-022-07905-0

[CR3] Adlakha YK, Saini N (2014) Brain microRNAs and insights into biological functions and therapeutic potential of brain enriched miRNA-128. Mol Cancer 13(1):1–18 24555688 10.1186/1476-4598-13-33 PMC3936914

[CR4] Agostini M, Tucci P, Steinert JR, Shalom-Feuerstein R, Rouleau M, Aberdam D, Concepcion CP (2011) microRNA-34a regulates neurite outgrowth, spinal morphology, and function. Proc Natl Acad Sci 108(52):21099–21104 22160706 10.1073/pnas.1112063108 PMC3248521

[CR5] Amoruso A, Blonda M, Gironi M, Grasso R, Di Francescantonio V, Scaroni F, Avolio C (2020) Immune and central nervous system-related miRNAs expression profiling in monocytes of multiple sclerosis patients. Sci Rep 10(1):6125 32273558 10.1038/s41598-020-63282-3 PMC7145856

[CR6] Aquino J, Bolontrade M, Garcia M, Podhajcer O, Mazzolini G (2010) Mesenchymal stem cells as therapeutic tools and gene carriers in liver fibrosis and hepatocellular carcinoma. Gene Ther 17(6):692–708 20220785 10.1038/gt.2010.10

[CR7] Aranda P, Agirre X, Ballestar E, Andreu EJ, Roman-Gomez J, Prieto I, Esteller M (2009) Epigenetic signatures associated with different levels of differentiation potential in human stem cells. PLoS ONE 4(11):e7809 19915669 10.1371/journal.pone.0007809 PMC2771914

[CR8] Arora S, Rana R, Chhabra A, Jaiswal A, Rani V (2013) miRNA–transcription factor interactions: a combinatorial regulation of gene expression. Mol Genet Genomics 288:77–87 23334784 10.1007/s00438-013-0734-z

[CR9] Baecher-Allan C, Kaskow BJ, Weiner HL (2018) Multiple sclerosis: mechanisms and immunotherapy. Neuron 97(4):742–768 29470968 10.1016/j.neuron.2018.01.021

[CR10] Barati S, Tahmasebi F, Faghihi F (2020) Effects of mesenchymal stem cells transplantation on multiple sclerosis patients. Neuropeptides 84:102095 33059244 10.1016/j.npep.2020.102095

[CR11] Barwari T, Joshi A, Mayr M (2016) MicroRNAs in cardiovascular disease. J Am Coll Cardiol 68(23):2577–2584 27931616 10.1016/j.jacc.2016.09.945

[CR12] Baulina N, Kulakova O, Kiselev I, Osmak G, Popova E, Boyko A, Favorova O (2018) Immune-related miRNA expression patterns in peripheral blood mononuclear cells differ in multiple sclerosis relapse and remission. J Neuroimmunol 317:67–76 29325906 10.1016/j.jneuroim.2018.01.005

[CR13] Blonda M, Amoruso A, Grasso R, Di Francescantonio V, Avolio C (2017) Multiple sclerosis treatments affect monocyte-derived microvesicle production. Front Neurol 8:422 28878732 10.3389/fneur.2017.00422 PMC5572278

[CR14] Boissart C, Nissan X, Giraud-Triboult K, Peschanski M, Benchoua A (2012) miR-125 potentiates early neural specification of human embryonic stem cells. Development 139(7):1247–1257 22357933 10.1242/dev.073627

[CR15] Brownlee WJ, Hardy TA, Fazekas F, Miller DH (2017) Diagnosis of multiple sclerosis: progress and challenges. Lancet 389(10076):1336–1346 27889190 10.1016/S0140-6736(16)30959-X

[CR16] Buhagiar A, Borg J, Ayers D (2020) Overview of current microRNA biomarker signatures as potential diagnostic tools for leukaemic conditions. Non-Coding RNA Res 5(1):22–26 10.1016/j.ncrna.2020.02.001 PMC7033436 32110743

[CR17] Camussi G, Deregibus MC, Bruno S, Cantaluppi V, Biancone L (2010) Exosomes/microvesicles as a mechanism of cell-to-cell communication. Kidney Int 78(9):838–848 20703216 10.1038/ki.2010.278

[CR18] Caplan A (2009) Why are MSCs therapeutic? New data: new insight. J Pathol: J Pathol Soc Great Britain Ireland 217(2):318–324 10.1002/path.2469 PMC8793150 19023885

[CR19] Carthew RW, Sontheimer EJ (2009) Origins and mechanisms of miRNAs and siRNAs. Cell 136(4):642–655 19239886 10.1016/j.cell.2009.01.035 PMC2675692

[CR20] Ceolotto G, Giannella A, Albiero M, Kuppusamy M, Radu C, Simioni P, Iori E (2017) miR-30c-5p regulates macrophage-mediated inflammation and pro-atherosclerosis pathways. Cardiovasc Res 113(13):1627–1638 29016810 10.1093/cvr/cvx157

[CR21] Chavakis E, Dimmeler S (2011) Homing of progenitor cells to ischemic tissues. Antioxid Redox Signal 15(4):967–980 20812875 10.1089/ars.2010.3582

[CR22] Chen C-Z, Li L, Lodish HF, Bartel DP (2004) MicroRNAs modulate hematopoietic lineage differentiation. Science 303(5654):83–86 14657504 10.1126/science.1091903

[CR23] Chen TS, Lai RC, Lee MM, Choo ABH, Lee CN, Lim SK (2010) Mesenchymal stem cell secretes microparticles enriched in pre-microRNAs. Nucleic Acids Res 38(1):215–224 19850715 10.1093/nar/gkp857 PMC2800221

[CR24] Cheng L-C, Pastrana E, Tavazoie M, Doetsch F (2009) miR-124 regulates adult neurogenesis in the subventricular zone stem cell niche. Nat Neurosci 12(4):399–408 19287386 10.1038/nn.2294 PMC2766245

[CR25] Choi YJ, Lin C-P, Ho JJ, He X, Okada N, Bu P, Chen C (2011) miR-34 miRNAs provide a barrier for somatic cell reprogramming. Nat Cell Biol 13(11):1353–1360 22020437 10.1038/ncb2366 PMC3541684

[CR26] Collino F, Deregibus MC, Bruno S, Sterpone L, Aghemo G, Viltono L, Camussi G (2010) Microvesicles derived from adult human bone marrow and tissue specific mesenchymal stem cells shuttle selected pattern of miRNAs. PLoS ONE 5(7):e11803 20668554 10.1371/journal.pone.0011803 PMC2910725

[CR27] Crop M, Baan C, Weimar W, Hoogduijn M (2009) Potential of mesenchymal stem cells as immune therapy in solid-organ transplantation. Transpl Int 22(4):365–376 19000235 10.1111/j.1432-2277.2008.00786.x

[CR28] Dai J, Su Y, Zhong S, Cong L, Liu B, Yang J, Jiang Y (2020) Exosomes: key players in cancer and potential therapeutic strategy. Signal Trans Target Ther 5(1):145 10.1038/s41392-020-00261-0 PMC7406508 32759948

[CR29] De Santis G, Ferracin M, Biondani A, Caniatti L, Tola MR, Castellazzi M, Fainardi E (2010) Altered miRNA expression in T regulatory cells in course of multiple sclerosis. J Neuroimmunol 226(1–2):165–171 20637509 10.1016/j.jneuroim.2010.06.009

[CR30] Dominici M, Le Blanc K, Mueller I, Slaper-Cortenbach I, Marini F, Krause D, Horwitz E (2006) Minimal criteria for defining multipotent mesenchymal stromal cells. the international society for cellular therapy position statement. Cytotherapy 8:315–317 16923606 10.1080/14653240600855905

[CR31] Duan Y, Luo Q, Wang Y, Ma Y, Chen F, Zhu X, Shi J (2020) Adipose mesenchymal stem cell-derived extracellular vesicles containing microRNA-26a-5p target TLR4 and protect against diabetic nephropathy. J Biol Chem 295(37):12868–12884 32580945 10.1074/jbc.RA120.012522 PMC7489897

[CR32] Ehya F, Tehrani HA, Garshasbi M, Nabavi SM (2017) Identification of miR-24 and miR-137 as novel candidate multiple sclerosis miRNA biomarkers using multi-staged data analysis protocol. Mol Biol Res Commun 6(3):127 29071282 10.22099/mbrc.2017.24861.1256 PMC5640895

[CR33] Essandoh K, Li Y, Huo J, Fan G-C (2016) MiRNA-mediated macrophage polarization and its potential role in the regulation of inflammatory response. Shock (augusta, Ga) 46(2):122 26954942 10.1097/SHK.0000000000000604 PMC4949115

[CR34] Faissner S, Plemel JR, Gold R, Yong VW (2019) Progressive multiple sclerosis: from pathophysiology to therapeutic strategies. Nat Rev Drug Discov 18(12):905–922 31399729 10.1038/s41573-019-0035-2

[CR35] Fayyad-Kazan H, Hamade E, Rouas R, Najar M, Fayyad-Kazan M, El Zein N, Al-Akoum C (2014) Downregulation of microRNA-24 and-181 parallels the upregulation of IFN-γ secreted by activated human CD4 lymphocytes. Hum Immunol 75(7):677–685 24704866 10.1016/j.humimm.2014.01.007

[CR36] Fenoglio C, Ridolfi E, Cantoni C, De Riz M, Bonsi R, Serpente M, Alvarez E (2013) Decreased circulating miRNA levels in patients with primary progressive multiple sclerosis. Mult Scler J 19(14):1938–1942 10.1177/1352458513485654 24277735

[CR37] Gallo S, Gili M, Lombardo G, Rossetti A, Rosso A, Dentelli P, Camussi G (2016) Stem cell-derived, microRNA-carrying extracellular vesicles: a novel approach to interfering with mesangial cell collagen production in a hyperglycaemic setting. PLoS ONE 11(9):e0162417 27611075 10.1371/journal.pone.0162417 PMC5017750

[CR38] Gandhi R, Healy B, Gholipour T, Egorova S, Musallam A, Hussain MS, Khoury S (2013) Circulating microRNAs as biomarkers for disease staging in multiple sclerosis. Ann Neurol 73(6):729–740 23494648 10.1002/ana.23880

[CR39] Gangaraju VK, Lin H (2009) MicroRNAs: key regulators of stem cells. Nat Rev Mol Cell Biol 10(2):116–125 19165214 10.1038/nrm2621 PMC4118578

[CR40] Gaudet AD, Fonken LK, Watkins LR, Nelson RJ, Popovich PG (2018) MicroRNAs: roles in regulating neuroinflammation. Neuroscientist 24(3):221–245 28737113 10.1177/1073858417721150 PMC8377730

[CR41] Ge X, Guo M, Hu T, Li W, Huang S, Yin Z, Kang C (2020) Increased microglial exosomal miR-124-3p alleviates neurodegeneration and improves cognitive outcome after rmTBI. Mol Ther 28(2):503–522 31843449 10.1016/j.ymthe.2019.11.017 PMC7001001

[CR42] Ghadiri N, Emamnia N, Ganjalikhani-Hakemi M, Ghaedi K, Etemadifar M, Salehi M, Nasr-Esfahani MH (2018) Analysis of the expression of mir-34a, mir-199a, mir-30c and mir-19a in peripheral blood CD4+ T lymphocytes of relapsing-remitting multiple sclerosis patients. Gene 659:109–117 29551498 10.1016/j.gene.2018.03.035

[CR43] Giacalone G, Clarelli F, Osiceanu A, Guaschino C, Brambilla P, Sorosina M, Rodegher M (2015) Analysis of genes, pathways and networks involved in disease severity and age at onset in primary-progressive multiple sclerosis. Mult Scler J 21(11):1431–1442 10.1177/1352458514564590 25583839

[CR44] Greco SJ, Rameshwar P (2007) MicroRNAs regulate synthesis of the neurotransmitter substance P in human mesenchymal stem cell-derived neuronal cells. Proc Natl Acad Sci 104(39):15484–15489 17855557 10.1073/pnas.0703037104 PMC2000543

[CR45] Greco SJ, Liu K, Rameshwar P (2007) Functional similarities among genes regulated by OCT4 in human mesenchymal and embryonic stem cells. Stem Cells 25(12):3143–3154 17761754 10.1634/stemcells.2007-0351

[CR46] Guerau-de-Arellano M, Smith KM, Godlewski J, Liu Y, Winger R, Lawler SE, Lovett-Racke AE (2011) Micro-RNA dysregulation in multiple sclerosis favours pro-inflammatory T-cell-mediated autoimmunity. Brain 134(12):3578–3589 22088562 10.1093/brain/awr262 PMC3235556

[CR47] Han X, Fan Z (2021) MicroRNAs regulation in osteogenic differentiation of mesenchymal stem cells. Front Dent Med. 10.3389/fdmed.2021.747068

[CR48] Haney MJ, Klyachko NL, Zhao Y, Gupta R, Plotnikova EG, He Z, Kabanov AV (2015) Exosomes as drug delivery vehicles for Parkinson’s disease therapy. J Control Release 207:18–30 25836593 10.1016/j.jconrel.2015.03.033 PMC4430381

[CR49] Hao Y, Miao J, Liu W, Cai K, Huang X, Peng L (2021) Mesenchymal stem cell-derived exosomes carry microRNA-125a to protect against diabetic nephropathy by targeting histone deacetylase 1 and downregulating endothelin-1. Diabet Metabol Syndr Obes: Target Ther 14:1405–1418 10.2147/DMSO.S286191 PMC8006976 33790607

[CR50] Hecker M, Thamilarasan M, Koczan D, Schröder I, Flechtner K, Freiesleben S, Zettl UK (2013) MicroRNA expression changes during interferon-beta treatment in the peripheral blood of multiple sclerosis patients. Int J Mol Sci 14(8):16087–16110 23921681 10.3390/ijms140816087 PMC3759901

[CR51] Heinrich E-M, Dimmeler S (2012) MicroRNAs and stem cells: control of pluripotency, reprogramming, and lineage commitment. Circ Res 110(7):1014–1022 22461365 10.1161/CIRCRESAHA.111.243394

[CR52] Hemmer B, Kerschensteiner M, Korn T (2015) Role of the innate and adaptive immune responses in the course of multiple sclerosis. Lancet Neurol 14(4):406–419 25792099 10.1016/S1474-4422(14)70305-9

[CR53] Hergenreider E, Heydt S, Tréguer K, Boettger T, Horrevoets AJ, Zeiher AM, Mayr M (2012) Atheroprotective communication between endothelial cells and smooth muscle cells through miRNAs. Nat Cell Biol 14(3):249–256 22327366 10.1038/ncb2441

[CR54] Hsia L-T, Ashley N, Ouaret D, Wang LM, Wilding J, Bodmer WF (2016) Myofibroblasts are distinguished from activated skin fibroblasts by the expression of AOC3 and other associated markers. Proc Natl Acad Sci 113(15):E2162–E2171 27036009 10.1073/pnas.1603534113 PMC4839407

[CR55] Hu R, Li H, Liu W, Yang L, Tan Y-F, Luo X-H (2010) Targeting miRNAs in osteoblast differentiation and bone formation. Expert Opin Ther Targets 14(10):1109–1120 20690886 10.1517/14728222.2010.512916

[CR56] Huang F, Zhu X, Hu X-Q, Fang Z-F, Tang L, Lu X-L, Zhou S-H (2013) Mesenchymal stem cells modified with miR-126 release angiogenic factors and activate notch ligand delta-like-4, enhancing ischemic angiogenesis and cell survival. Int J Mol Med 31(2):484–492 23229021 10.3892/ijmm.2012.1200

[CR57] Humphreys BD, Valerius MT, Kobayashi A, Mugford JW, Soeung S, Duffield JS, Bonventre JV (2008) Intrinsic epithelial cells repair the kidney after injury. Cell Stem Cell 2(3):284–291 18371453 10.1016/j.stem.2008.01.014

[CR58] Ieda M, Fu J-D, Delgado-Olguin P, Vedantham V, Hayashi Y, Bruneau BG, Srivastava D (2010) Direct reprogramming of fibroblasts into functional cardiomyocytes by defined factors. Cell 142(3):375–386 20691899 10.1016/j.cell.2010.07.002 PMC2919844

[CR59] Jagot F, Davoust N (2016) Is it worth considering circulating microRNAs in multiple sclerosis? Front Immunol 7:129 27092141 10.3389/fimmu.2016.00129 PMC4821089

[CR60] Jakob P, Landmesser U (2012) Role of microRNAs in stem/progenitor cells and cardiovascular repair. Cardiovasc Res 93(4):614–622 22135162 10.1093/cvr/cvr311

[CR61] Jernås M, Malmeström C, Axelsson M, Nookaew I, Wadenvik H, Lycke J, Olsson B (2013) MicroRNA regulate immune pathways in T-cells in multiple sclerosis (MS). BMC Immunol 14(1):1–11 23895517 10.1186/1471-2172-14-32 PMC3734042

[CR62] Jiang Y, Jahagirdar BN, Reinhardt RL, Schwartz RE, Keene CD, Ortiz-Gonzalez XR, Blackstad M (2002) Pluripotency of mesenchymal stem cells derived from adult marrow. Nature 418(6893):41–49 12077603 10.1038/nature00870

[CR63] Jin J, Shi Y, Gong J, Zhao L, Li Y, He Q, Huang H (2019) Exosome secreted from adipose-derived stem cells attenuates diabetic nephropathy by promoting autophagy flux and inhibiting apoptosis in podocyte. Stem Cell Res Ther 10:1–15 30876481 10.1186/s13287-019-1177-1 PMC6419838

[CR64] Jovičić A, Roshan R, Moisoi N, Pradervand S, Moser R, Pillai B, Luthi-Carter R (2013) Comprehensive expression analyses of neural cell-type-specific miRNAs identify new determinants of the specification and maintenance of neuronal phenotypes. J Neurosci 33(12):5127–5137 23516279 10.1523/JNEUROSCI.0600-12.2013 PMC6705001

[CR65] Judson RL, Babiarz JE, Venere M, Blelloch R (2009) Embryonic stem cell–specific microRNAs promote induced pluripotency. Nat Biotechnol 27(5):459–461 19363475 10.1038/nbt.1535 PMC2743930

[CR66] Junker A, Krumbholz M, Eisele S, Mohan H, Augstein F, Bittner R, Meinl E (2009) MicroRNA profiling of multiple sclerosis lesions identifies modulators of the regulatory protein CD47. Brain 132(12):3342–3352 19952055 10.1093/brain/awp300

[CR67] Kacperska MJ, Jastrzebski K, Tomasik B, Walenczak J, Konarska-Krol M, Glabinski A (2015) Selected extracellular microRNA as potential biomarkers of multiple sclerosis activity—preliminary study. J Mol Neurosci 56:154–163 25487315 10.1007/s12031-014-0476-3 PMC4382531

[CR68] Kim H, Lee G, Ganat Y, Papapetrou EP, Lipchina I, Socci ND, Studer L (2011) miR-371-3 expression predicts neural differentiation propensity in human pluripotent stem cells. Cell Stem Cell 8(6):695–706 21624813 10.1016/j.stem.2011.04.002

[CR69] Kimura K, Hohjoh H, Fukuoka M, Sato W, Oki S, Tomi C, Yamamura T (2018) Circulating exosomes suppress the induction of regulatory T cells via let-7i in multiple sclerosis. Nat Commun 9(1):17 29295981 10.1038/s41467-017-02406-2 PMC5750223

[CR70] Lakshmipathy U, Hart RP (2008) Concise review: microRNA expression in multipotent mesenchymal stromal cells. Stem Cells 26(2):356–363 17991914 10.1634/stemcells.2007-0625 PMC2673465

[CR71] Leri A, Kajstura J, Anversa P (2011) Role of cardiac stem cells in cardiac pathophysiology: a paradigm shift in human myocardial biology. Circ Res 109(8):941–961 21960726 10.1161/CIRCRESAHA.111.243154 PMC3299091

[CR72] Li Z, Yang CS, Nakashima K, Rana TM (2011) Small RNA-mediated regulation of iPS cell generation. EMBO J 30(5):823–834 21285944 10.1038/emboj.2011.2 PMC3049216

[CR73] Liang X, Zhang L, Wang S, Han Q, Zhao RC (2016) Exosomes secreted by mesenchymal stem cells promote endothelial cell angiogenesis by transferring miR-125a. J Cell Sci 129(11):2182–2189 27252357 10.1242/jcs.170373

[CR74] Lindberg RL, Hoffmann F, Mehling M, Kuhle J, Kappos L (2010) Altered expression of miR-17-5p in CD4+ lymphocytes of relapsing–remitting multiple sclerosis patients. Eur J Immunol 40(3):888–898 20148420 10.1002/eji.200940032

[CR75] Liu C, Teng Z-Q, Santistevan NJ, Szulwach KE, Guo W, Jin P, Zhao X (2010) Epigenetic regulation of miR-184 by MBD1 governs neural stem cell proliferation and differentiation. Cell Stem Cell 6(5):433–444 20452318 10.1016/j.stem.2010.02.017 PMC2867837

[CR76] Liu J, Wang L, Su Z, Wu W, Cai X, Li D, Pan G (2014) A reciprocal antagonism between miR-376c and TGF-β signaling regulates neural differentiation of human pluripotent stem cells. FASEB J 28(11):4642–4656 25114173 10.1096/fj.13-249342

[CR77] Ma X, Zhou J, Zhong Y, Jiang L, Mu P, Li Y, Nagarkatti P (2014) Expression, regulation and function of microRNAs in multiple sclerosis. Int J Med Sci 11(8):810 24936144 10.7150/ijms.8647 PMC4057480

[CR78] Mao R, Shen J, Hu X (2020) BMSCs-derived exosomal microRNA-let-7a plays a protective role in diabetic nephropathy via inhibition of USP22 expression. Life Sci 268:118937–118937 33347877 10.1016/j.lfs.2020.118937

[CR79] Meoli EM, Oh U, Grant CW, Jacobson S (2011) TGF-β signaling is altered in the peripheral blood of subjects with multiple sclerosis. J Neuroimmunol 230(1–2):164–168 21093933 10.1016/j.jneuroim.2010.10.028 PMC4988390

[CR80] Mizuno Y, Yagi K, Tokuzawa Y, Kanesaki-Yatsuka Y, Suda T, Katagiri T, Amemiya T (2008) miR-125b inhibits osteoblastic differentiation by down-regulation of cell proliferation. Biochem Biophys Res Commun 368(2):267–272 18230348 10.1016/j.bbrc.2008.01.073

[CR81] Mohammed EM (2020) Environmental influencers, microRNA, and multiple sclerosis. J Cent Nerv Sys Dis 12:1179573519894955 10.1177/1179573519894955 PMC6971968 32009827

[CR82] Moore CS, Rao VT, Durafourt BA, Bedell BJ, Ludwin SK, Bar-Or A, Antel JP (2013) miR-155 as a multiple sclerosis–relevant regulator of myeloid cell polarization. Ann Neurol 74(5):709–720 23818336 10.1002/ana.23967

[CR83] Murugaiyan G, Beynon V, Mittal A, Joller N, Weiner HL (2011) Silencing microRNA-155 ameliorates experimental autoimmune encephalomyelitis. J Immunol 187(5):2213–2221 21788439 10.4049/jimmunol.1003952 PMC3167080

[CR84] O’Rourke JR, Georges SA, Seay HR, Tapscott SJ, McManus MT, Goldhamer DJ, Harfe BD (2007) Essential role for Dicer during skeletal muscle development. Dev Biol 311(2):359–368 17936265 10.1016/j.ydbio.2007.08.032 PMC2753295

[CR85] Pauley KM, Cha S, Chan EK (2009) MicroRNA in autoimmunity and autoimmune diseases. J Autoimmun 32(3–4):189–194 19303254 10.1016/j.jaut.2009.02.012 PMC2717629

[CR86] Peng L, Chen Y, Shi S, Wen H (2022) Stem cell-derived and circulating exosomal microRNAs as new potential tools for diabetic nephropathy management. Stem Cell Res Ther 13(1):25 35073973 10.1186/s13287-021-02696-w PMC8785577

[CR87] Peter ME (2009) Let-7 and miR-200 microRNAs: guardians against pluripotency and cancer progression. Cell Cycle 8(6):843–852 19221491 10.4161/cc.8.6.7907 PMC2688687

[CR88] Phinney DG, Prockop DJ (2007) Concise review: mesenchymal stem/multipotent stromal cells: the state of transdifferentiation and modes of tissue repair—current views. Stem Cells 25(11):2896–2902 17901396 10.1634/stemcells.2007-0637

[CR89] Pöllinger B (2012) IL-17 producing T cells in mouse models of multiple sclerosis and rheumatoid arthritis. J Mol Med 90:613–624 22231742 10.1007/s00109-011-0841-4

[CR90] Ponomarev ED, Veremeyko T, Weiner HL (2013) MicroRNAs are universal regulators of differentiation, activation, and polarization of microglia and macrophages in normal and diseased CNS. Glia 61(1):91–103 22653784 10.1002/glia.22363 PMC3434289

[CR91] Prada I, Gabrielli M, Turola E, Iorio A, D’Arrigo G, Parolisi R, Lombardi M (2018) Glia-to-neuron transfer of miRNAs via extracellular vesicles: a new mechanism underlying inflammation-induced synaptic alterations. Acta Neuropathol 135:529–550 29302779 10.1007/s00401-017-1803-x PMC5978931

[CR92] Raposo G, Stoorvogel W (2013) Extracellular vesicles: exosomes, microvesicles, and friends. J Cell Biol 200(4):373–383 23420871 10.1083/jcb.201211138 PMC3575529

[CR93] Reijerkerk A, Lopez-Ramirez MA, van Het Hof B, Drexhage JA, Kamphuis WW, Kooij G, Horrevoets AJ (2013) MicroRNAs regulate human brain endothelial cell-barrier function in inflammation: implications for multiple sclerosis. J Neurosci 33(16):6857–6863 23595744 10.1523/JNEUROSCI.3965-12.2013 PMC6618888

[CR94] Rosa A, Brivanlou AH (2011) A regulatory circuitry comprised of miR-302 and the transcription factors OCT4 and NR2F2 regulates human embryonic stem cell differentiation. EMBO J 30(2):237–248 21151097 10.1038/emboj.2010.319 PMC3025464

[CR95] Rustad KC, Gurtner GC (2012) Mesenchymal stem cells home to sites of injury and inflammation. Adv Wound Care 1(4):147–152 10.1089/wound.2011.0314 PMC3623614 24527296

[CR96] Schratt GM, Tuebing F, Nigh EA, Kane CG, Sabatini ME, Kiebler M, Greenberg ME (2006) A brain-specific microRNA regulates dendritic spine development. Nature 439(7074):283–289 16421561 10.1038/nature04367

[CR97] Shah A, Meese E, Blin N (2010) Profiling of regulatory microRNA transcriptomes in various biological processes: a review. J Appl Genet 51:501–507 21063068 10.1007/BF03208880

[CR98] Siegel SR, Mackenzie J, Chaplin G, Jablonski NG, Griffiths L (2012) Circulating microRNAs involved in multiple sclerosis. Mol Biol Rep 39:6219–6225 22231906 10.1007/s11033-011-1441-7

[CR99] Sievers C, Meira M, Hoffmann F, Fontoura P, Kappos L, Lindberg RL (2012) Altered microRNA expression in B lymphocytes in multiple sclerosis: towards a better understanding of treatment effects. Clin Immunol 144(1):70–79 22659298 10.1016/j.clim.2012.04.002

[CR100] Smalheiser NR (2007) Exosomal transfer of proteins and RNAs at synapses in the nervous system. Biol Direct 2(1):1–15 18053135 10.1186/1745-6150-2-35 PMC2219957

[CR101] Smirnova L, Gräfe A, Seiler A, Schumacher S, Nitsch R, Wulczyn FG (2005) Regulation of miRNA expression during neural cell specification. Eur J Neurosci 21(6):1469–1477 15845075 10.1111/j.1460-9568.2005.03978.x

[CR102] Smith KM, Guerau-de-Arellano M, Costinean S, Williams JL, Bottoni A, Mavrikis Cox G, Lovett-Racke AE (2012) miR-29ab1 deficiency identifies a negative feedback loop controlling Th1 bias that is dysregulated in multiple sclerosis. J Immunol 189(4):1567–1576 22772450 10.4049/jimmunol.1103171 PMC3411895

[CR103] Søndergaard HB, Hesse D, Krakauer M, Sørensen PS, Sellebjerg F (2013) Differential microRNA expression in blood in multiple sclerosis. Mult Scler J 19(14):1849–1857 10.1177/1352458513490542 23773985

[CR104] Sospedra, M., & Martin, R. (2016). Immunology of multiple sclerosis*.* Paper presented at the Seminars in neurology. 10.1055/s-0036-1579739 27116718

[CR105] Stampanoni Bassi M, Iezzi E, Buttari F, Gilio L, Simonelli I, Carbone F, Furlan R (2020) Obesity worsens central inflammation and disability in multiple sclerosis. Mult Scler J 26(10):1237–1246 10.1177/1352458519853473 31161863

[CR106] Subramanyam D, Lamouille S, Judson RL, Liu JY, Bucay N, Derynck R, Blelloch R (2011) Multiple targets of miR-302 and miR-372 promote reprogramming of human fibroblasts to induced pluripotent stem cells. Nat Biotechnol 29(5):443–448 21490602 10.1038/nbt.1862 PMC3685579

[CR107] Sun J, Zhao F, Zhang W, Lv J, Lv J, Yin A (2018) BMSC s and miR-124a ameliorated diabetic nephropathy via inhibiting notch signalling pathway. J Cell Mol Med 22(10):4840–4855 30024097 10.1111/jcmm.13747 PMC6156290

[CR108] Suzuki Y, Kim HW, Ashraf M, Haider HK (2010) Diazoxide potentiates mesenchymal stem cell survival via NF-κB-dependent miR-146a expression by targeting Fas. Am J Physiol-Heart Circ Physiol 299(4):H1077–H1082 20656888 10.1152/ajpheart.00212.2010 PMC2957349

[CR109] Svahn AJ, Giacomotto J, Graeber MB, Rinkwitz S, Becker TS (2016) miR-124 Contributes to the functional maturity of microglia. Dev Neurobiol 76(5):507–518 26184457 10.1002/dneu.22328

[CR110] Tahmasebi F, Barati S (2023) The role of microglial depletion approaches in pathological condition of CNS. Cell Mol Neurobiol. 10.1007/s10571-023-01326-8 36738403 10.1007/s10571-023-01326-8 PMC11410134

[CR111] Takahashi K, Yamanaka S (2006) Induction of pluripotent stem cells from mouse embryonic and adult fibroblast cultures by defined factors. Cell 126(4):663–676 16904174 10.1016/j.cell.2006.07.024

[CR112] Tarassishin L, Loudig O, Bauman A, Shafit-Zagardo B, Suh HS, Lee SC (2011) Interferon regulatory factor 3 inhibits astrocyte inflammatory gene expression through suppression of the proinflammatory miR-155 and miR-155. Glia 59(12):1911–1922 22170100 10.1002/glia.21233 PMC3241213

[CR113] Tata PR, Tata NR, Kühl M, Sirbu IO (2011) Identification of a novel epigenetic regulatory region within the pluripotency associated microRNA cluster. EEmiRC Nucleic Acids Res 39(9):3574–3581 21247880 10.1093/nar/gkq1344 PMC3089473

[CR114] Tay YM-S, Tam W-L, Ang Y-S, Gaughwin PM, Yang H, Wang W, Perera RJ (2008) MicroRNA-134 modulates the differentiation of mouse embryonic stem cells, where it causes post-transcriptional attenuation of Nanog and LRH1. Stem Cells 26(1):17–29 17916804 10.1634/stemcells.2007-0295

[CR115] Tsai MS, Hwang SM, Chen KD, Lee YS, Hsu LW, Chang YJ, Chao AS (2007) Functional network analysis of the transcriptomes of mesenchymal stem cells derived from amniotic fluid, amniotic membrane, cord blood, and bone marrow. Stem Cells 25(10):2511–2523 17556597 10.1634/stemcells.2007-0023

[CR116] Viñas JL, Spence M, Gutsol A, Knoll W, Burger D, Zimpelmann J, Burns KD (2018) Receptor-ligand interaction mediates targeting of endothelial colony forming cell-derived exosomes to the kidney after ischemic injury. Sci Rep 8(1):16320 30397255 10.1038/s41598-018-34557-7 PMC6218514

[CR117] Viswanathan SR, Daley GQ, Gregory RI (2008) Selective blockade of microRNA processing by Lin28. Science 320(5872):97–100 18292307 10.1126/science.1154040 PMC3368499

[CR118] Wang J, Park JW, Drissi H, Wang X, Xu R-H (2014) Epigenetic regulation of miR-302 by JMJD1C inhibits neural differentiation of human embryonic stem cells. J Biol Chem 289(4):2384–2395 24318875 10.1074/jbc.M113.535799 PMC3900981

[CR119] Wang B, Yao K, Huuskes BM, Shen H-H, Zhuang J, Godson C, Ricardo SD (2016a) Mesenchymal stem cells deliver exogenous microRNA-let7c via exosomes to attenuate renal fibrosis. Mol Ther 24(7):1290–1301 27203438 10.1038/mt.2016.90 PMC5088767

[CR120] Wang H, Ye Y, Zhu Z, Mo L, Lin C, Wang Q, Lu G (2016b) MiR-124 regulates apoptosis and autophagy process in MPTP model of Parkinson’s disease by targeting to B im. Brain Pathol 26(2):167–176 25976060 10.1111/bpa.12267 PMC8029438

[CR121] Waschbisch A, Atiya M, Linker RA, Potapov S, Schwab S, Derfuss T (2011) Glatiramer acetate treatment normalizes deregulated microRNA expression in relapsing remitting multiple sclerosis. PLoS ONE 6(9):e24604 21949733 10.1371/journal.pone.0024604 PMC3174971

[CR122] Wei W, Ao Q, Wang X, Cao Y, Liu Y, Zheng SG, Tian X (2021) Mesenchymal stem cell-derived exosomes: a promising biological tool in nanomedicine. Front Pharmacol 11:590470 33716723 10.3389/fphar.2020.590470 PMC7944140

[CR123] Xu N, Papagiannakopoulos T, Pan G, Thomson JA, Kosik KS (2009) MicroRNA-145 regulates OCT4, SOX2, and KLF4 and represses pluripotency in human embryonic stem cells. Cell 137(4):647–658 19409607 10.1016/j.cell.2009.02.038

[CR124] Xu Q, Seeger FH, Castillo J, Iekushi K, Boon RA, Farcas R, Zeiher AM (2012) Micro-RNA-34a contributes to the impaired function of bone marrow-derived mononuclear cells from patients with cardiovascular disease. J Am Coll Cardiol 59(23):2107–2117 22651868 10.1016/j.jacc.2012.02.033

[CR125] Xu Z, Dong D, Chen X, Huang H, Wen S (2015) MicroRNA-381 negatively regulates TLR4 signaling in A549 cells in response to LPS stimulation. BioMed Res Int. 10.1155/2015/849475 26688820 10.1155/2015/849475 PMC4672107

[CR126] Xu J, Zheng Y, Wang L, Liu Y, Wang X, Li Y, Chi G (2022) miR-124: a promising therapeutic target for central nervous system injuries and diseases. Cell Mol Neurobiol 42(7):2031–2053 33886036 10.1007/s10571-021-01091-6 PMC11421642

[CR127] Yaldizli Ö, Putzki N (2009) Natalizumab in the treatment of multiple sclerosis. Ther Adv Neurol Disord 2(2):115–128 21180646 10.1177/1756285608101861 PMC3002624

[CR128] Yang D, Li T, Wang Y, Tang Y, Cui H, Tang Y, Le W (2012) miR-132 regulates the differentiation of dopamine neurons by directly targeting Nurr1 expression. J Cell Sci 125(7):1673–1682 22328530 10.1242/jcs.086421

[CR129] Yang D, Wang W-Z, Zhang X-M, Yue H, Li B, Lin L, Fu J (2014) MicroRNA expression aberration in Chinese patients with relapsing remitting multiple sclerosis. J Mol Neurosci 52:131–137 24217794 10.1007/s12031-013-0138-x

[CR130] Yoo AS, Staahl BT, Chen L, Crabtree GR (2009) MicroRNA-mediated switching of chromatin-remodelling complexes in neural development. Nature 460(7255):642–646 19561591 10.1038/nature08139 PMC2921580

[CR131] Zhang J, Cheng Y, Cui W, Li M, Li B, Guo L (2014) MicroRNA-155 modulates Th1 and Th17 cell differentiation and is associated with multiple sclerosis and experimental autoimmune encephalomyelitis. J Neuroimmunol 266(1–2):56–63 24332164 10.1016/j.jneuroim.2013.09.019

[CR132] Zhao C, Sun G, Li S, Shi Y (2009) A feedback regulatory loop involving microRNA-9 and nuclear receptor TLX in neural stem cell fate determination. Nat Struct Mol Biol 16(4):365–371 19330006 10.1038/nsmb.1576 PMC2667220

[CR133] Zhao T, Sun F, Liu J, Ding T, She J, Mao F, Yan Y (2019) Emerging role of mesenchymal stem cell-derived exosomes in regenerative medicine. Curr Stem Cell Res Ther 14(6):482–494 30819086 10.2174/1574888X14666190228103230

[CR134] Zhu S, Pan W, Song X, Liu Y, Shao X, Tang Y, Liu W (2012) The microRNA miR-23b suppresses IL-17-associated autoimmune inflammation by targeting TAB2, TAB3 and IKK-α. Nat Med 18(7):1077–1086 22660635 10.1038/nm.2815

